# MicroRNAs and Bone Metastasis: A New Challenge

**DOI:** 10.3390/molecules190710115

**Published:** 2014-07-11

**Authors:** Martine Croset, Daniele Santini, Michele Iuliani, Marco Fioramonti, Alice Zoccoli, Bruno Vincenzi, Giuseppe Tonini, Francesco Pantano

**Affiliations:** 1 Institut national de la santé et de la recherche médicale (INSERM), UMR 1033, Lyon F-69008, France; E-Mail: martine.croset@inserm.fr; 2 Medical Oncology, Campus Bio-Medico University of Rome, Rome 00128, Italy; E-Mails: m.iuliani@unicampus.it (M.I.); m.fioramonti@unicampus.it (M.F.); a.zoccoli@unicampus.it (A.Z.); b.vincenzi@unicampus.it (B.V.); g.tonini@unicampus.it (G.T.); f.pantano@unicampus.it (F.P.)

**Keywords:** bone metastases, microRNAs, epithelial-mesenchymal transition, osteomimicry

## Abstract

The development of bone metastases requires multistep and multicellular machinery consisting not only of processes shared with any type of metastases (formation of a pre-metastatic niche, chemotaxis of tumor cells into the host tissue, tumor cells escape from the microvasculature), but also biological interactions that are strictly related to the particular bone microenvironment (bone marrow colonization by cancer cells, osteomimicry, deregulation of bone homeostasis). MiRNAs are highly conserved, small RNAs molecules that regulate gene expression. The functional consequence of miRNA deregulation lies in the mRNA targets whose expression is altered. MiRNA networks acting as upstream regulators of these genes interfere with the initial steps of tumor local invasion and cancer cell intravasation, mainly by regulating the epithelial-mesenchymal transition, the motility, invasiveness and survival abilities of these cells. The miRNA-mediated regulation on the steps of bone tropism, floatage, homing and finally bone colonization is more tissue specific, being dependent on the expression pattern of target miRNAs in bone marrow sinusoids, bone cells and microenvironment. In that, miRNA specific expression signatures that can distinguish between primary tumors from their corresponding bone metastases might be determinants of clinical aggressiveness. In this review, we focus on the current advances on functions and molecular mechanisms by which miRNAs exert their biological roles in regulating bone metastases development.

## 1. Introduction

Bone metastases are a common place complication of several types of cancers, including breast, prostate and lung cancer [1]. The occurrence of these bone metastases deeply impairs the prognosis and quality of life of patients and is responsible for significant morbidity (bone pain, pathological fractures, nerve compression, hypercalcemia). Bone metastases are often osteolytic (due to significant bone destruction), sometimes sclerotic (due to an excess of bone formation) or mixed [2]. Indeed, tumor cells secrete factors that may disrupt physiological bone remodeling processes through the deregulation of the normal osteoclast and osteoblast functions. For example, osteolysis is the result of stimulation by tumor cells of osteoclast activity and inhibition of osteoblast, while an opposite phenomenon occurs during osteosclerosis. Moreover, the mineralized bone matrix plays an important role in metastasis formation acting as a reservoir of growth factors and calcium that, once released from the matrix during degradation, can have a mitogenic effect on the tumor cells, thus leading to a vicious cycle where the resorption/bone formation and tumor proliferation feed off each other. The complex sequence of events that lead to the onset of bone metastases not only involve processes common to any other metastasis (invasion of blood vessels from the primary tumor, establishing of a pre-metastatic niche in the host tissue, chemotaxis of tumor cells into the host tissue, extravasation of tumor cells from blood vessels) but also processes that are more specific to the bone tissue (bone marrow colonization, osteomimicry, deregulation of osteoblast/osteoclast activity) [3]. All steps of this sequence are regulated by multiple factors and molecular pathways, mainly through the tight control of genes expressed by interacting cells [4,5,6]. The small non-coding microRNAs (miRNAs) which have the capacity to regulate multiple genes are master regulators of gene expression and thus redirect or reprogram biological pathways [7]. They also are capable of controlling the aberrant biological activities characteristic of tumor cells [8]; consequently, in cancer, they can act either as promoters or suppressors of tumor development and metastatic progression [7,9,10,11].

## 2. Bone Marrow Colonization

It has been described that about 30% of breast cancer patients may harbor bone marrow micrometastases at the time of primary cancer diagnosis [12], but half of them will never develop clinically established metastases. Furthermore, patients with breast and prostate cancer may remain metastasis free even for decades after surgical removal of the primary cancer. Indeed, according to Paget’s “seed and soil theory”, metastatic cells can enter into bone marrow and remain alive and start to proliferate or remain in a quiescent status for many years. Preclinical evidences suggest that metastasis arises from a small fraction of cells with the capacity to self-renew and to differentiate to specialized cell types with limited proliferative potential, often termed cancer stem cells (CSCs) [13,14]. CSCs are proposed to acquire a great part of their biological features through undergoing the so called epithelial-mesenchymal transition (EMT). EMT is a biologic process that allows a polarized epithelial cell, which normally interacts with basement membrane via its basal surface to undergo multiple biochemical changes that enable it to assume a mesenchymal cell phenotype. During EMT, transformation of normal epithelial cells disrupts cell-cell and cell extracellular matrix contacts and migrate to other locations in the body [15] providing these early cancer cells with the capacity to infiltrate the surrounding tissue and ultimately metastasize to distant sites [16]. Once these cells reach the bone marrow, they interact with anatomical entities in contact with the bone called “niches”. Two different niches exist: “endosteal niche” where stem cells are closely associated with spindle-shaped N-cadherin-positive osteoblasts (SNO) involved in the maintenance of stem cells quiescence and “vascular niche” within central marrow, where more differentiated hematopoietic cells generally are located [17]. Bone metastatic disseminated tumor cells (DTCs) target the niches where hematopoietic stem cells (HSCs) reside and compete with them for occupancy. Indeed, DTCs and HSCs are believed to use similar mechanisms to gain access to the HSC niche. After entering the niche, DTCs are likely to evict HSCs into the peripheral blood or drive them into progenitor pools. In the competition for the HSC niche, PCa cells may directly and indirectly drive HSC maturity, therefore vacating the niche. As a result of the competition with DTCs, more hematopoietic progenitor cells (HPCs) are found circulating in the peripheral blood of patients with metastatic PCa than either patients with localized PCa or healthy age-matched controls. Under normal physiologic conditions, HSCs are believed to reside mainly within the bone marrow HSC niche, while some HSCs are known to leave the marrow, differentiate into HPCs, and circulate throughout the body. However, the question as to whether HPCs mobilized by DTCs are functionally the same as normal HPCs remains unanswered [18]. Several receptors and their ligands have been implicated in these intercellular interactions; for example CXCR-4 (receptor on HSC)/SDF-1 (ligand secreted by osteoblastic lineage cells) axis represents one of the most extensively investigated while hyaluronan and osteopontin (ligands expressed in the bone)/CD44 (receptor and putative marker for CSC) binding suggest a possible adhesive interaction for circulating tumor cell arrest. Moreover these steps involved in cancer metastasis development have been shown to be orchestrated by pleiotropically acting molecules—transcription factors and miRNAs. Several transcription factors, including Snail [19], Slug [20], Twist [21], ZEB1, and ZEB2 [22], have been identified as inducers of EMT and tumor metastasis. More recently, miR-200s and miR-205 family [23,24], miR-143 and miR-145 [25,26], let-7 [27], miR-203 [28], miR-34a [29] have emerged as new epithelial markers and repressors of EMT and stem cell properties (Table 1). 

### 2.1. miR-200s and miR-205 Family

Oncogenic EMT is programmed by transcription factors such as Snail, Slug and Twist, which are potent E-cadherin suppressors, and is stimulated by TGFb. The miR-200 family and miR-205 are crucial regulators of EMT/MET by targeting ZEB1 and ZEB2 mRNAs [23,24]. MiRNAs that target CSCs affect the self-renewal and differentiation capacities of these cells and impair metastatic progression. Let-7 miRNAs which expression is consistently downregulated in metastatic cancer cells [30], inhibit cell stemness and breast cancer metastasis through silencing of MYC, Ras and HMGA2 [27], while miR-200c inhibits cell stemness and metastasis by targeting ZEBs, TGFb and BMI1 [31]. Furthermore, miRNAs regulate the expression of genes which have been reported as pro- or anti-metastatic in one or several steps of metastatic dissemination to the bone: the gain in enhanced migratory capacity and invasiveness, the production of ECM components, angiogenesis and elevated resistance to apoptosis and the bone homing and colonization [32]. The miR-200 family has also been shown to promote metastatic colonization at distal sites by altering the tumor secretome through direct targeting of Sec23 homolog A (SEC23A) [8]. It should also be noticed that experimental evidence suggest that interference with the microenvironmental support impairs the *de novo* formation of bone metastases *in vivo* [33]. miRNAs produced in the bone microenvironment might interfere with bone metastasis. Indeed, miRNAs can be transported via gap-junctions or exosomes from bone marrow stroma to breast cancer cells and modulate the dormancy of DTCs [34]. Although still confusing, increasing evidences demonstrated how tumor-associated microenvironment can produce exosomes and both cancer cell-derived or stromal cell-derived exosomes are thus able to alter the tumor environment and may participate in forming a distant metastatic niche to promote metastasis [35,36]. Regarding gap-junctions, the exchange of miRNA through those between the bone marrow stroma and breast cancer cells may be an important factor that contributes to the dormancy of the cancer cells to impart a quiescent phenotype in bone marrow. The gap-junctions identified between bone marrow stroma and breast cancer cells in the endosteal region is consistent with the failure of high-dose chemotherapy in autologous hematopoietic stem cell transplantation [37].

**Table 1:** List of principal miRNAs involved in bone metastases development.

	miRNA	Function	References
**EMT/Stemness**	miR-200s family; miR-205	Suppressor	Paterson *et al.*, 2008 [23]; Gregory *et al.*, 2008 [24];
	Let-7 family	Suppressor	Yu *et al.*, 2007 [27];
	miR-10b	Activator	Zhao *et al.*, 2012; Ma *et al.*, 2010 [38,39];
	miR-143; miR-145	Suppressor	Peng *et al.*, 2011; Guo *et al.*, 2013 [25,26];
	miR-100	Suppressor	Wang *et al.*, 2014 [40];
	miR-203	Suppressor	Saini *et al.*, 2011 [28];
	miR-34a	Suppressor	Liu *et al.*, 2011 [29];
	miR-7	Suppressor	Okuda *et al.*, 2013 [41];
**Osteomimicry**	miR-30s family	Suppressor	Croset *et al.*, 2011 [42];
	miR-218	Activator/Suppressor	Tie *et al.*, 2010 [43];
	miR-204; miR-211; miR-379	Suppressor of osteoclast function	Pollari *et al.*, 2012 [44];
**Bone Remodelling**	miR-33a	Suppressor of osteoclast function	Kuo *et al.*, 2013 [45];
	miR-326	Activator of osteoclast function	Valencia *et al.*, 2013 [46]

### 2.2. miR-10b

Approximately 50% of metastasis-positive patients had elevated miR-10b levels in their primary tumors when compared to metastasis-free patients [38]. It has been demonstrated in animal model that overexpression of mir-10b in non-metastatic breast tumor cell promoted dissemination of tumor cells in the lungs. Indeed transcription factor Twist directly induced miR-10b expression which, in turn, inhibited the expression of the transcription factor homeobox D10 (*HOXD10*). Interestingly, Twist1, which has been reported as a link between EMT and stemness in breast carcinomas [38], is expressed in disseminated breast cancer cells that persist in the bone marrow after chemotherapy. Moreover serum miR-10b concentrations were found to be significantly higher in breast cancer patients with bone metastases than in patients without bone metastases, suggesting that the Twist-induced miR-10b expression could be also involved in bone metastasis formation [39]. The potential of miR-10b as target for anti-metastatic therapy has been therefore studied in animal model of breast cancer metastasis [39]. In this study, miR-10b antagonist was efficiently delivered to rapidly growing tumor cells and it prevented metastasis dissemination to secondary organs without marks of cytotoxicity. 

### 2.3. miR-143, miR-145

Comparison of array-based miRNA profiles of human primary prostate tumors and matched bone metastasis showed that expression levels of miR-145, -143, -33a, -100 and -508-5p were the most highly down-regulated in bone metastasis specimens [25]. Ectopic expression of miR-143 and miR-145 in the aggressive and androgen-insensitive PC-3 prostate cancer cell line reduced the migratory and invasive capacities of these tumor cells *in vitro* and their propensity to metastasize to bone *in vivo*. The expression of the mesenchymal markers, fibronectin and vimentin, was also decreased suggesting a direct involvement in modulating EMT [25]. In a retrospective investigation of 12 patients without bone metastasis and 10 patients with bone metastasis the expression levels of these two miRNAs were significantly lower in bone metastatic patients. Moreover miRs-143 and -145 expression levels were inversely correlated to free PSA and the Gleason score [25]. These findings suggest that miR-143 and miR-145 could be used as biomarkers in discriminating different stages of prostate carcinomas and predicting bone metastasis.

Recently it has been demonstrated that AGO2 was a direct target of miR-100 which is involved in regulation of bone metastatic process in prostate cancer. In particular downregulation of AGO2 by miR-100 repressed migration, invasion, EMT and stemness of prostate cancer cells. Furthermore, miR-100 expression was negatively correlated with bone metastasis of prostate cancer patients [40]. 

### 2.4. miR-203

An antimetastatic role for miR-203 in prostate cancer progression and bone metastasis has been reported [28]. The expression level of miR-203 in human prostate adenocarcinoma is lower than in normal adjacent tissue. Low miR-203 expression correlates with advanced clinicopathological stage and high Gleason score in patients with prostate cancer. In addition, miR-203 expression is down-regulated in prostate cancer cell lines derived from bone metastasis (PC3, MDA-PCa-2b and VCaP cells). Ectopic expression of miR-203 significantly decreases the metastatic capacity of PC-3 cells in a mouse model of bone metastasis. The phenotype of miR-203-transfected cells (marked morphological changes, from fibroblasts to epithelial-like phenotype, and reduced migration/invasion) is reminiscent of a reverse transition from an EMT to a mesenchymal-to-epithelial state (MET). Importantly, miR-203 can pleiotropically regulate a cohort of metastatic effectors that includes apart from ZEB2, Bmi1 and survivin, involved in the development of hormone resistance in PCa and associated with unfavorable outcome. Finally miR-203 regulates bone-specific effectors including Runx2, a master regulator of bone metastasis [28]. 

### 2.5. miR-34a/miR-7

miR-34a has been identified as a potent inhibitor of prostate CSCs and metastasis by directly repressing CD44 [29]. The authors purified CSC population from prostate cancer xenograft models. These CD44-positive CSCs harbor enhanced clonogenicity, tumor-initiating and metastatic properties and are also depleted in miR-34a expression, compared to CD44-negative CSCs. Restoring miR-34a expression inhibits clonogenic expansion, tumor development and metastasis by targeting CD44 expression. Similarly, Okuda *et al.* [41] reported that CD44-expressing CSCs isolated from MDA-MB-231 breast cancer cell subpopulations which were highly metastatic to brain and bone expressed low levels of miR-7 and that this miRNA modulated KLF4, one of the essential genes for induced pluripotent stem cell. Forced expression of miR-7 suppressed the ability of CSCs to metastasize to brain but not to bone. Thus, the miRNA-mediated regulation on gene expression seems to be not only cell specific (CSCc *vs.* non CSCs) but also tissue specific (bone *vs.* brain).

## 3. Osteomimicry

It has been postulated that tumor cells which spread in the bone marrow must then adapt to the bone environment for the subsequent development of overt metastasis. It is probably for this reason that the DTCs that are in the bone marrow remain for several years in a state of quiescence before suddenly be reactivated and start to grow. This adaptation requires that tumor cells begin to express genes that are normally expressed by bone cells. This process is called osteomimicry. For example, immunohistochemical analysis of samples of primary tumor and matched metastases (liver, lung, bone) of breast cancer patients showed that only metastatic cells express proteins of bone marrow origin (cathepsin K, Integrin Beta 3 osteonectin, cadherin-11, connexin-43 and Runx2) [47,48].

### 3.1. miR-30s Family

It has been demonstrated that the miRNA-30s are expressed at higher levels in hormone-dependent and well differentiated breast tumors, compared the levels observed in hormone-refractory tumors [49]. miRNA-30s have been reported acting as a tumor suppressor in lung [50], breast [51] and anaplasic thyroid [52] cancers, mostly by regulating EMT. Their lower expression levels in lymph node metastases *vs.* primary tumors suggests a role for miR-30s during metastatic dissemination of breast carcinomas [53]. Moreover miRNA-30s regulate osteoblast differentiation [53] leading to the hypothesis of their involvement in regulating cancer cell osteomimetic phenotype. Indeed differential miRNAs profiling expression in breast cancer cell lines MDA-MB-231 and B02 (subpopulation of the parental MDA-MB-231 cell line that has been selected for its high propensity to metastasize to bone in murine models [54]) that the expression level of each of the members of the miRNA-30 family (miRNA-30a,b,c,d,e) is downregulated within MDA-BO2 cells compared to parental cell line [42]. Restoring the expression of the miRNA-30s in MDA-BO2 cells decreased significantly bone metastasis progression in mice mainly targeting osteomimetic genes like connective tissue growth factor (CTGF), connexin 43, integrinβ3 (ITGb3), cadherin-11, and Runx2 were associated to osteomimicry of MDA-B02 cells.

### 3.2. miR-218

Regulation of osteomimicry-related tumor activity has also been reported for miR-218 [43]. Mir-218 appears as an “osteo-miR” by controlling bone formation through the upregulaton of Runx2 and the downregulation of Wnt signaling inhibitors (sclerostin, dickkopf-2 and secreted frizzled-related protein2) in osteoblasts. It also appears as a pro-metastatic miRNA through stimulation of the expression of Wnt-related proteins (bone sialoprotein and osteopontin) in metastatic breast cancer cells. In addition miR-218 may facilitate MDA-MB-231 breast cancer bone metastasis by up-regulating CXCR4, a chemokine receptor supporting breast cancer cell migration to bone and mediating tumor growth in bone. Although miR-218 regulates positively the molecular mechanisms underlying bone metastasis formation it appears as tumor suppressor in other types of cancers [43], pointing out that its biological activity depends on the cellular microenvironment.

## 4. Deregulation of Osteoblast/Osteoclast Activity

Bone lesions result in the interaction between cancer cells, bone microenvironment and bone cells themselves. The tumor cells use bone-derived growth factors involved in the coupling between osteoclasts/osteoblasts to promote their own development. In turn, they secrete factors (PTHrP, IL-1, IL-6, IL-8, IL-11, M-CSF) that act in a paracrine fashion to activate osteoclast-mediated bone resorption. In addition, cancer cells can release other growth factors that can promote (ET1, IGFs) or inhibit (DKK1, Noggin, sclerostin) osteoblast activity. These processes are accompanied by the release of growth factors and cytokines (TGFb, IGFs) embedded in the matrix during bone formation. These molecules released from the resorbed bone matrix act in turn on cancer cells to promote their proliferation [55,56,57].

### 4.1. miR-204, miR-211, miR-379

TGFb is involved in the vicious cycle between bone and tumor cells. It is released from bone in the microenvironment during bone resorption and it stimulates cancer cells to produce pro-osteolytic factors such as IL-11. To identify miRNAs that potentially regulate this process, Pollari *et al.* [44] profiled the miRNA expression in the MDA-MB-231 cell line and a highly bone metastatic variant and found that miRNAs were differently expressed between the two cell lines. Among these miRNAs, the authors identify 3 miRNAs, miR-204, miR-211 and miR-379, as potent inhibitors of TGFb-induced IL-11 secretion. In addition, gene expression analysis showed that miR-204 and miR-379 downregulated a set of genes involved in TGFb signalling.

### 4.2. miR-33a and miR-336

It has been reported that PTHrP, is a direct target of miR-33a in lung cancer. miR-33a levels are inversely correlated with PTHrP expression between human normal bronchial cell line and lung cancer cell lines. Restoring miR-33a expression reduces the stimulatory effect of lung cancer cells on the production of osteoclastogenesis activator RANKL, (receptor activator of nuclear factor kappa-B ligand) and M-CSF (macrophage colony-stimulating factor) on osteoblasts, while the expression of PTHrP and IL-8 are decreased [45]. Thus a low miR-33a expression contributes to cancer-mediated bone destruction and may even predict a poor prognosis for lung cancer patients. Indeed, miRNAs are promising candidates as prognosis markers in cancer in regard of their high stability and their accessibility in serum patients. In this respect, Valencia *et al.* [46] assessed the validity of measuring serum miRNAs in a murine model of human lung cancer bone metastasis and compared miRNA serum levels with those of standard biochemical markers of bone turnover such as PINP (procollagen I amino-terminal propeptide), BGP (osteocalcin) and CTX (carboxyterminal telopeptide). Using this model of lung cancer, PINP (procollagen I amino-terminal propeptide) exhibited a strong correlation with osteolytic lesions and tumor burden at early and late stages of bone colonization. In contrast, BGP and CTX demonstrated a strong correlation only at late stages. Interestingly, in mice model serum levels of miR-326 strongly associated with tumor burden and PINP in vehicle-treated animals, whereas no association was found in animals treated with an inhibitor of bone resorption (zoledronate). These results suggest that miR-326 could serve as a biochemical marker for monitoring bone metastatic progression in advanced lung cancer. 

## 5. Clinical Usefulness of miRNAs in Bone Metastatic Cancer

MiRNAs expression represents a specific pattern for individual tissues and both miRNA family and levels differ between cancer and normal tissues, constituting the so-called “miRNA signature” [58]. MiRNAs are stable in different biological fluids such as plasma, urine, saliva, seminal, amniotic and pleural effusions and for this reason they are considered as good diagnostic and prognostic markers and predictors of chemotherapeutic response. In particular, was found that miRNAs are very useful to perform an accurate diagnosis and therapy for breast and prostate cancer that highly metastatize to the bone [59].

In breast carcinomas, aberrant concentrations of miRNAs in serum correlate disease progression and metastatic spread. Patients with lymph node-positive breast cancer and those with node-negative are characterized by different plasma levels of miR-10b and miR-373 [60]. Increased concentrations of miR-373 were also associated with HER2 negative status of the primary tumor. Also hormone receptor status could be detected by miRNA signature, because miR-17 is deregulated in hormone receptor-positive patients, whereas serum levels of miR-34a, miR-21, miR-126, miR-155, miR-199a and miR-335 are deregulated in hormone receptor-negative patient. In addition, patients with breast cancer with advanced-stage disease had significantly more miR-34a in their blood stream than patients at early stages of the disease, and changes in serum levels of miR-34a together with miR-10b and miR-155 correlated with the presence of metastases [59].

Urine levels miR-107 and miR-574-3p were significantly higher in men with prostate cancer than in healthy men. Metastatic patients exhibit plasma levels of miR-21, miR-221 and particularly miR-141 significantly higher than patient with locally advanced-stage disease [59].

MiR-141, but also miR-375 and miR-378, were significantly overexpressed in patient with castration-resistant prostate cancer (CRPC), whereas expression of miR-409-3p was reduced. Also hormone- dependence status could be monitored through miRNA signature, with serum levels of miR-21 in patient with hormone-refractory prostate cancer that were higher than in patients with androgen-dependent and localized prostate cancer. Finally plasma levels of miR-20a differentiate in patients with stage III tumours from stage I and II [59].

The future potential of miRNA-based therapy is in its infancy: while the effectiveness of specific miRNAs in metastatic bone disease is still under investigation. Pre-clinical mouse studies have demonstrated promising results in targeting the metastatic tumor cells and a Phase I clinical trial of miR-34 replacement therapy is currently underway [61].

## 6. Conclusions

Recent advances have shown the important role played by adaptation of metastatic cells in the bone environment and the subsequent crosstalk between tumour and host tissue, underlining their involvement in skeletal metastasis growth. It is still not clear whether cancer cells already possess an osteomimetic phenotype when they detach from the primary tumour or whether these characteristics are instead acquired when they colonize the bone niche. Anyway it has been demonstrated that CSCs are able to localize in new sites where the “soil” can exert an important role on their fate and that cancer cells need a biological signature to invade bone. Analysis of this bone-specific metastasis-signatures in primary carcinomas has provided biological insight into the mechanisms driving metastasis and identified bone-specific metastasis genes. The miRNA-mediated regulation on these gene expression leads to a cascade of events that alter gene interaction networks at different steps of bone metastasis progression. In this, miRNAs are reported as general regulators of tumor progression, metastatic dissemination and immune invasion whereas their activity in interfering with bone colonization lies more specifically in the mRNA targets whose expression is altered in bone metastasis. Uncovering the genes under the control of these miRNAs and how they integrate into the bone metastatic gene interaction network will further help our understanding of the disease and lead to new therapy. To accomplish this, identifying the genome-wide targets of miRNAs and transferring their bone metastasis-suppressive activity from bench into clinical settings for predictive biomarkers and/or treatment of bone lesions seem essential. Although the miRNA-based therapeutic approach to interfere with bone metastasis is an attractive one, it remains puzzling in regard of the large numbers of transcripts, sometimes with opposing functional consequences, that could be targeted by a single miRNA. The efficiency and safe delivery of agents such as miRNA mimics or antagonist to a growing metastatic tumour or to already-established metastasis need to further studied.

## References

[1] Yin J.J., Pollock C.B., Kelly K. (2005). Mechanisms of cancer metastasis to the bone. Cell Res..

[2] Suva L.J., Washam C., Nicholas R.W., Griffin R.J. (2011). Bone metastasis: Mechanisms and therapeutic opportunities. Nat. Rev. Endocrinol..

[3] Weilbaecher K.N., Guise T.A., McCauley L.K. (2011). Cancer to bone: A fatal attraction. Nat. Rev. Cancer.

[4] Nguyen D.X., Massagué J. (2007). Genetic determinants of cancer metastasis. Nat. Rev. Genet..

[5] Smid M., Wang Y., Klijn J.G., Sieuwerts A.M., Zhang Y., Atkins D., Martens J.W., Foekens J.A. (2006). Genes associated with breast cancer metastatic to bone. J. Clin. Oncol..

[6] Van’t Veer L.J., Dai H., van de Vijver M.J., He Y.D., Hart A.A., Mao M., Peterse H.L., van der Kooy K., Marton M.J., Witteveen A.T. (2002). Gene expression profiling predicts clinical outcome of breast cancer. Nature.

[7] Bartel D.P. (2004). MicroRNAs: Genomics, biogenesis, mechanism, and function. Cell.

[8] Browne G., Taipaleenmäki H., Stein G.S., Stein J.L., Lian J.B. (2014). MicroRNAs in the control of metastatic bone disease. Trends Endocrinol. MeTable.

[9] Iorio M.V., Croce C.M. (2012). MicroRNA involvement in human cancer. Carcinogenesis.

[10] Profumo V., Gandellini P. (2013). MicroRNAs: Cobblestones on the road to cancer metastasis. Crit. Rev. Oncog..

[11] Wang L., Wang J. (2012). MicroRNA-mediated breast cancer metastasis: From primary site to distant organs. Oncogene.

[12] Pantel K., Alix-Panabières C., Riethdorf S. (2009). Cancer micrometastases. Nat. Rev. Clin. Oncol..

[13] Al-Hajj M., Wicha M.S., Benito-Hernandez A., Morrison S.J., Clarke M.F. (2003). ProspectiveIdentification of tumorigenic breast cancer cells. Proc. Natl. Acad. Sci. USA.

[14] Ricci-Vitiani L., Lombardi D.G., Pilozzi E., Biffoni M., Todaro M., Peschle C., de Maria R. (2007). Identification and expansion of human colon-cancer initiating cells. Nature.

[15] Turley E.A., Veiseh M., Radisky D.C., Bissell M.J. (2008). Mechanisms of disease: Epithelial-mesenchymal transition-does cellular plasticity fuel neoplastic progression?. Nat. Clin. Pract. Oncol..

[16] Radisky D.C., LaBarge M.A. (2008). Epithelial-mesenchymal transition and the stem cell phenotype. Cell Stem Cell.

[17] Yin T., Li L. (2006). The stem cell niches in bone. J. Clin. Investig..

[18] Shiozawa Y., Pedersen E.A., Havens A.M., Jung Y., Mishra A., Joseph J., Kim J.K., Patel L.R., Ying C., Ziegler A.M. (2011). Human prostate cancer metastases target the hematopoietic stem cell niche to establish footholds in mouse bone marrow. J. Clin. Investig..

[19] Cano A., Pérez-Moreno M.A., Rodrigo I., Locascio A., Blanco M.J., del Barrio M.G., Portillo F., Nieto M.A. (2000). The transcription factor snail controls epithelial-mesenchymal transitions by repressing E-cadherin expression. Nat. Cell Biol..

[20] Hajra K.M., Chen D.Y., Fearon E.R. (2002). The SLUG zinc-finger protein represses E-cadherin in breast cancer. Cancer Res..

[21] Yang J., Mani S.A., Donaher J.L., Ramaswamy S., Itzykson R.A., Come C., Savagner P., Gitelman I., Richardson A., Weinberg R.A. (2004). Twist, a master regulator of morphogenesis, plays an essential role in tumor metastasis. Cell.

[22] Eger A., Aigner K., Sonderegger S., Dampier B., Oehler S., Schreiber M., Berx G., Cano A., Beug H., Foisner R. (2005). DeltaEF1 is a transcriptional repressor of E-cadherin and regulates epithelial plasticity in breast cancer cells. Oncogene.

[23] Paterson E.L., Kolesnikoff N., Gregory P.A., Bert A.G., Khew-Goodall Y., Goodall G.J. (2008). The microRNA-200 family regulates epithelial to mesenchymal transition. Sci. World J..

[24] Gregory P.A., Bert A.G., Paterson E.L., Barry S.C., Tsykin A., Farshid G., Vadas M.A., Khew-Goodall Y., Goodall G.J. (2008). The miR-200 family and miR-205 regulate epithelial to mesenchymal transition by targeting ZEB1 and SIP1. Nat. Cell Biol..

[25] Peng X., Guo W., Liu T., Wang X., Tu X., Xiong D., Chen S., Lai Y., Du H., Chen G. (2011). Identification of miRs-143 and -145 that is associated with bone metastasis of prostate cancer and involved in the regulation of EMT. PLoS One.

[26] Guo W., Ren D., Chen X., Tu X., Huang S., Wang M., Song L., Zou X., Peng X. (2013). HEF1 promotes epithelial mesenchymal transition and bone invasion in prostate cancer under the regulation of microRNA-145. J. Cell. Biochem..

[27] Yu F., Yao H., Zhu P., Zhang X., Pan Q., Gong C., Huang Y., Hu X., Su F., Lieberman J. (2007). let-7 regulates self renewal and tumorigenicity of breast cancer cells. Cell.

[28] Saini S., Majid S., Yamamura S., Tabatabai L., Suh S.O., Shahryari V., Chen Y., Deng G., Tanaka Y., Dahiya R. (2011). Regulatory Role of mir-203 in Prostate Cancer Progression and Metastasis. Clin. Cancer Res..

[29] Liu C., Kelnar K., Liu B., Chen X., Calhoun-Davis T., Li H., Patrawala L., Yan H., Jeter C., Honorio S. (2011). The microRNA miR-34a inhibits prostate cancer stem cells and metastasis by directly repressing CD44. Nat. Med..

[30] Vimalraj S., Miranda P.J., Ramyakrishna B., Selvamurugan N. (2013). Regulation of breast cancer and bone metastasis by microRNAs. Dis. Markers.

[31] Brabletz S., Brabletz T. (2010). The ZEB/miR-200 feedback loop—A motor of cellular plasticity in development and cancer?. EMBO Rep..

[32] Abraham B.K., Fritz P., McClellan M., Hauptvogel P., Athelogou M., Brauch H. (2005). Prevalence of CD44+/CD24-/low cells in breast cancer may not be associated with clinical outcome but may favor distant metastasis. Clin. Cancer Res..

[33] Van der Pluijm G., Que I., Sijmons B., Buijs J.T., Löwik C.W., Wetterwald A., Thalmann G.N., Papapoulos S.E., Cecchini M.G. (2005). Interference with the microenvironmental support impairs the de novo formation of bone metastases *in vivo*. Cancer Res..

[34] Lim P.K., Bliss S.A., Patel S.A., Taborga M., Dave M.A., Gregory L.A., Greco S.J., Bryan M., Patel P.S., Rameshwar P. (2011). Gap junction-mediated import of microRNA from bone marrow stromal cells can elicit cell cycle quiescence in breast cancer cells. Cancer Res..

[35] Hoffman R.M. (2013). Stromal-cell and cancer-cell exosomes leading the metastatic exodus for the promised niche. Breast Cancer Res..

[36] Luga V., Zhang L., Viloria-Petit A.M., Ogunjimi A.A., Inanlou M.R., Chiu E., Buchanan M., Hosein A.N., Basik M., Wrana J.L. (2012). Exosomes mediate stromal mobilization of autocrine Wnt-PCP signaling in breast cancer cell migration. Cell.

[37] Gregory L.A., Ricart R.A., Patel S.A., Lim P.K., Rameshwar P. (2011). microRNAs, Gap Junctional intercellular communication and mesenchymal stem cells in breast cancer metastasis. Curr. Cancer Ther. Rev..

[38] Zhao F.L., Hu G.D., Wang X.F., Zhang X.H., Zhang Y.K., Yu Z.S. (2012). Serum overexpression of microRNA-10b in patients with bone metastatic primary breast cancer. J. Int. Med. Res..

[39] Ma L., Reinhardt F., Pan E., Soutschek J., Bhat B., Marcusson E.G., Teruya-Feldstein J., Bell G.W., Weinberg R.A. (2010). Therapeutic silencing of miR-10b inhibits metastasis in a mouse mammary tumor model. Nat. Biotechnol..

[40] Wang M., Ren D., Guo W., Wang Z., Huang S., Du H., Song L., Peng X. (2014). Loss of miR-100 enhances migration, invasion, epithelial-mesenchymal transition and stemness properties in prostate cancer cells through targeting Argonaute 2. Int. J. Oncol..

[41] Okuda H., Xing F., Pandey P.R., Sharma S., Watabe M., Pai S.K., Mo Y.Y., Iiizumi-Gairani M., Hirota S., Liu Y. (2013). miR-7 suppresses brain metastasis of breast cancer stem-like cells by modulating KLF4. Cancer Res..

[42] Croset M., Bachelier R., Allioli N., Hong S.-S., Agami R., Clézardin P. (2011). Targeting breast cancer osteomimetic genes by miRNA-30s decreases the formation of metastatic osteolytic lesions in mice. Bull. Cancer.

[43] Tie J., Pan Y., Zhao L., Wu K., Liu J., Sun S., Guo X., Wang B., Gang Y., Zhang Y. (2010). MiR-218 inhibits invasion and metastasis of gastric cancer by targeting the Robo1 receptor. PLoS Genet..

[44] Pollari S., Leivonen S.K., Perälä M., Fey V., Käkönen S.M., Kallioniemi O. (2012). Identification of microRNAs inhibiting TGF-β-induced IL-11 production in bone metastatic breast cancer cells. PLoS One.

[45] Kuo P.L., Liao S.H., Hung J.Y., Huang M.S., Hsu Y.L. (2013). MicroRNA-33a functions as a bone metastasis suppressor in lung cancer by targeting parathyroid hormone related protein. Biochim.Biophys. Acta.

[46] Valencia K., Martín-Fernández M., Zandueta C., Ormazábal C., Martínez-Canarias S., Bandrés E., de la Piedra C., Lecanda F. (2013). miR-326 associates with biochemical markers of bone turnover in lung cancer bone metastasis. Bone.

[47] Le Gall C., Bellahcène A., Bonnelye E., Gasser J.A., Castronovo V., Green J., Zimmermann J., Clézardin P. (2007). A cathepsin K inhibitor reduces breast cancer induced osteolysis and skeletal tumor burden. Cancer Res..

[48] Bellahcène A., Bachelier R., Detry C., Lidereau R., Clézardin P., Castronovo V. (2007). Transcriptome analysis reveals an osteoblast-like phenotype for human osteotropic breast cancer cells. Breast Cancer Res. Treat..

[49] Iorio M.V., Ferracin M., Liu C.G., Veronese A., Spizzo R., Sabbioni S., Magri E., Pedriali M., Fabbri M., Campiglio M. (2005). MicroRNA gene expression deregulation in human breast cancer. Cancer Res..

[50] Kumarswamy R., Mudduluru G., Ceppi P., Muppala S., Kozlowski M., Niklinski J., Papotti M., Allgayer H. (2012). MicroRNA-30a inhibits epithelial-to-mesenchymal transition by targeting Snai1 and is downregulated in non-small cell lung cancer. Int. J. Cancer.

[51] Zhang N., Wang X., Huo Q., Sun M., Cai C., Liu Z., Hu G., Yang Q. (2013). MicroRNA-30a suppresses breast tumor growth and metastasis by targeting metadherin. Oncogene.

[52] Visone R., Pallante P., Vecchione A., Cirombella R., Ferracin M., Ferraro A., Volinia S., Coluzzi S., Leone V., Borbone E. (2007). Specific microRNAs are downregulated in human thyroid anaplastic carcinomas. Oncogene.

[53] Wu T., Zhou H., Hong Y., Li J., Jiang X., Huang H. (2012). miR-30 family members negatively regulate osteoblast differentiation. J. Biol. Chem..

[54] Peyruchaud O., Winding B., Pécheur I., Serre C.M., Delmas P., Clézardin P. (2001). Early detection of bone metastases in a murine model using fluorescent human breast cancer cells: Application to the use of the bisphosphonate zoledronic acid in the treatment of osteolytic lesions. J. Bone Miner. Res..

[55] Santini D., Pantano F., Vincenzi B., Tonini G., Bertoldo F. (2012). The role of bone microenvironment, vitamin D and calcium. Recent Results Cancer Res..

[56] Santini D., Galluzzo S., Vincenzi B., Schiavon G., Fratto E., Pantano F., Tonini G. (2007). New developments of aminobisphosphonates: The double face of Janus. Ann. Oncol..

[57] Zoccoli A., Iuliani M., Pantano F., Imperatori M., Intagliata S., Vincenzi B., Marchetti P., Papapietro N., Denaro V., Tonini G. (2012). Premetastatic niche: Ready for new therapeutic interventions?. Expert Opin. Ther. Targets.

[58] Volinia S., Calin G.A., Liu C.G., Ambs S., Cimmino A., Petrocca F., Visone R., Iorio M., Roldo C., Ferracin M. (2006). A microRNA expression signature of human solid tumors defines cancer gene targets. Proc. Natl. Acad. Sci. USA.

[59] Schwarzenbach H., Nishida N., Calin G.A., Pantel K. (2014). Clinical relevance of circulating cell-free microRNAs in cancer. Nat. Rev. Clin. Oncol..

[60] Roth C., Rack B., Müller V., Janni W., Pantel K., Schwarzenbach H. (2010). Circulating microRNAs as blood-based markers for patients with primary and metastatic breast cancer. Breast Cancer Res..

[61] Bouchie A. (2013). First microRNA mimic enters clinic. Nat. Biotechnol..

